# Mindfulness acting with awareness and emotional eating among polycystic ovary syndrome women with infertility: the mediating role of depression

**DOI:** 10.3389/fpsyg.2024.1499705

**Published:** 2024-12-11

**Authors:** Mengye Yang, Xiaoyu Wang, Yan Zhang, Weina Qian, Yan Tang

**Affiliations:** ^1^Reproductive Center, Suzhou Municipal Hospital, Suzhou, China; ^2^Institute of Nursing, Nanjing Medical University, Nanjing, China

**Keywords:** polycystic ovary syndrome, mindfulness, emotional eating, depression, infertile

## Abstract

Emotional eating, characterized by the tendency to increase food intake in response to negative emotional states, is often linked to poor emotion regulation. While mindfulness-based interventions have been studied for their benefits in reducing emotional eating, less is known about how inherent mindfulness traits, relate to emotional regulation particularly among individuals with polycystic ovary syndrome (PCOS), a population known for high rates of psychological distress and disordered eating behaviors. This study investigates the associations between different facets of mindfulness, depressive symptoms, and emotional eating among individuals with PCOS and infertility who had not received any formal mindfulness intervention. A cross-sectional study was conducted involving 334 individuals. Participants completed the Five Facet Mindfulness Questionnaire-Short Form (FFMQ-SF), the Patient Health Questionnaire (PHQ-9) to assess depressive symptoms, and the Dutch Eating Behavior Questionnaire (DEBQ) to measure emotional eating. Structural Equation Modeling (SEM) was employed to examine the relationships between inherent mindfulness traits (i.e., observation, describing, acting with awareness, non-reactivity, and non-judgment), depressive symptoms, anxiety, and emotional eating. The findings indicated that the trait of acting with awareness may reduce emotional eating through its influence on depressive symptoms, while observational mindfulness was found to be associated with increased emotional eating without prior external mindfulness training. In conclusion, Mindfulness is a multidimensional construct, with its facets contributing differently to emotional regulation and eating behaviors in individuals with PCOS and infertility. Future research should explore these nuances to develop more targeted interventions.

## Introduction

Polycystic ovary syndrome (PCOS) is characterized by a series of symptoms, including irregular menstrual cycles, excess androgen levels, and ovarian cysts. It is a common endourological disorder that influence about 8–13% individuals in their reproductive years (World Health Organization, 2023). Polycystic ovary syndrome (PCOS) is a leading cause of infertility among individuals. The incidence of infertility among individuals with PCOS varies but is generally quite high. Research indicates that approximately 70 to 80% of individuals with PCOS experience infertility issues (Melo et al., 2015). The primary reason for this high rate is anovulation, where the ovaries do not release an oocyte during a menstrual cycle, which is a common symptom of PCOS. Other factors such as insulin resistance, obesity, and hormonal imbalances further contribute to infertility in individuals with PCOS (Layacha and Biswas, 2023). What’s more, PCOS also has potential long-term effects on health, such as increased risk of type 2 diabetes and cardiovascular disease (Layacha and Biswas, 2023; Stener-Victorin et al., 2024). Individuals diagnosed with PCOS show higher prevalence of self-reported psychological issues, such as anxiety, depression and reduced levels of body satisfaction (Dybciak et al., 2023; Teede et al., 2023). A meta-analysis reported about 34% of individuals with PCOS reported depressive symptoms and 43% reported anxiety symptoms while general infertile individuals show prevalence of 20% for anxiety and depression (Wang et al., 2021).

Emotional eating is a significant issue for individuals with PCOS. A systematic review conducted in 2023 found that the incidence of emotional eating ranges from 30 to 50% among individuals with PCOS. Emotional eating is defined as eating in response to an emotional state rather than hunger, often as a form of emotional comfort or as a way to manage negative feelings in unhealthy ways (Burnatowska et al., 2023). It is related to key health concerns such as disordered eating and obesity, which can potentially lower the success rates of assisted reproductive technologies. Furthermore, several studies have found that maternal eating disorders increase the risk of gestational diabetes mellitus (GDM) during pregnancy and emotional disorders in childhood (Kimmel et al., 2016; Silvani et al., 2020). Research indicates that individuals with PCOS are at high risk for emotional eating, often originating during adolescence (Steegers-Theunissen et al., 2020). This is primarily due to the physical manifestations of the disease, such as hirsutism, acne, acanthosis nigricans, and obesity, which can lead to body image disturbances and depression. Additionally, prolonged menstrual irregularities and infertility in adulthood exacerbate their psychological burden. A longitudinal study tracking adolescent and young adult females over 4 years found that early adverse eating behaviors and negative life experiences can contribute to the development of emotional eating, which may progress into eating disorders (Steegers-Theunissen et al., 2020).

Negative emotional experiences have critical influence on eating behavior. This is supported by the growing number of studies indicating that the emotional eating is associated with depression and anxiety (Ouwens et al., 2009; Dakanalis et al., 2023). High levels of anxiety or depression cause chronic stress, which activates the Hypothalamic–Pituitary–Adrenal (HPA) axis, increases cortisol secretion, and inhibits the release of dopamine and serotonin. This neurotransmitter imbalance is often compensated for through food intake, leading to emotional eating (Chang et al., 2022). Individuals store these learned experiences in cognitive patterns, which makes it difficult for them to distinguish between hunger and anxiety, leading to compulsive overeating (Ziauddeen et al., 2015). Emotional eating not only reflects an individual’s susceptibility to emotional influences in their eating behaviors but also indicates a lack of emotional regulation, perception, and coping skills when dealing with emotional distress (Macht and Simons, 2011). This lack of emotional management makes individuals more vulnerable to their emotions. Previous study have supported the Escape theories which posit that the emotional eating may avert attention away from distressing stimuli and thereby rely on the avoidance distraction (Aldao et al., 2010). Therefore, it is important for individual not only to notice their physical and emotional feelings, but also express and being open to these feelings that contributes to the unhealthy consummatory.

The ability to focus on emotional reactions, perceptual experiences, and bodily sensations is an important aspect of mindfulness. Mindfulness is defined as a mental state achieved by focusing one’s awareness on the present moment while calmly acknowledging and accepting one’s feelings, thoughts, and bodily sensations (Kabat-Zinn, 1982). Although everyone inherently possesses this mindfulness trait, it can be enhanced through mindfulness interventions. Indeed, one of the main functions of mindfulness is to regulate emotions by targeting poor emotion regulation, low self-efficacy, and positive emotionality (Baroni et al., 2018; Polizzi and Lynn, 2021). Specifically, mindfulness enhances an individual’s capacity to manage and respond to emotional experiences in a balanced and adaptive manner. For example, mindfulness practices have been associated with reduced emotional reactivity and increased emotional clarity, allowing individuals to respond to stressors with greater composure and less impulsivity (Chambers et al., 2009). This improved emotional regulation can lead to better mental health outcomes and increased resilience in the face of challenges. Since emotional eating may be driven by maladaptive affect regulation, numerous researchers have focused on the role of mindfulness interventions in addressing eating disorders, particularly emotional eating. Studies have demonstrated that mindfulness is negatively associated with emotional and uncontrolled eating, as well as with constructs related to emotional eating (Gouveia et al., 2019; Czepczor-Bernat et al., 2020). However, across these studies, mindfulness has generally been operationalized in a broad sense rather than considering the specific facets of trait mindfulness.

To better understand the mechanism of mindfulness in psychological treatment, Bishop et al. (2004) established an operational definition that precisely describes the various components of mindfulness, including observing, describing, acting with awareness, non-judgment, and non-reactivity (Bishop et al., 2004). Following this, the Monitor and Acceptance Theory (MAT) was proposed, explaining the effects of mindfulness on affective and health outcomes through two active mechanisms: attention monitoring and acceptance. According to MAT, acceptance is an umbrella term encompassing a broad range of constructs, including the non-judging and non-reactivity facets of mindfulness (Lindsay and Creswell, 2017). Since acceptance involves early engagement with and disengagement from negative experiences, it reduces emotional reactivity and the need for maladaptive emotional regulation strategies. Recently, a cross-sectional study conducted in a community sample examined the effects of specific components of mindfulness on negative emotions and emotional eating. The results showed that depressive symptoms significantly interacted with non-judging of inner experience to predict emotional eating (Hsu and Forestell, 2021); individuals high in non-judging reported less emotional eating compared to those average or low in non-judging. Additionally, another study involving 259 individuals indicated that acting with awareness and non-reactive mindfulness might be important treatment (Barnhart et al., 2021).

These studies highlight the importance of the different components of mindfulness in regulating emotions and eating behavior. Specifically, non-judgment, acting with awareness, and non-reactivity are key regulatory factors when dealing with psychological issues such as depressive symptoms and emotional eating.

Investigating how individuals with higher levels of trait mindfulness, particularly specific facets such as acting with awareness, naturally engage in mindful eating could provide valuable insights. Such research could inform the development of mindfulness-based eating interventions, enhancing their accessibility and effectiveness by focusing on the natural cultivation of mindfulness without the need for intensive training. Therefore, a cross-sectional study was conducted to examine the associations among trait mindfulness facets (including observation, describing, acting with awareness, non-reactivity, non-judgement), negative emotions and emotional eating with individuals with PCOS and infertility who had not received any formal mindfulness intervention. We hypothesized that mindfulness facets, particularly observation, acting with awareness and non-judgement may have effect on emotional eating through their influence on symptoms of depression/anxiety. Higher levels of mindfulness lead to lower levels of depression, which in turn reduces emotional eating.

## Method

### Study population

This study was conducted at a reproductive center in Suzhou, China, from April 2023 to July 2024. A total of 334 individuals who visited the outpatient department and were diagnosed with infertility (diagnosed as a couple unable to conceive after 1 year of regular, with unprotected intercourse) and PCOS (according to Rotterdam criteria, where two of the three were met: oligo-ovulation or anovulation, clinical manifestations of hyperandrogenism and/or hyperandrogenemia, or ovarian polycystic changes) were invited to participate in this study. Previously eligible patients were not contacted by phone. The inclusion criteria required participants to be above the age of 18 years, and express willingness to provide written informed consent. Considering that this study assessed individuals’ mindfulness traits using a trait-based scale, rather than applying a mindfulness-based intervention, it aimed to explore how inherent mindfulness traits are associated with depression, anxiety, and emotional eating. And since prior studies have indicated that mindfulness practice may influence emotional regulation and eating behaviors (Alberts et al., 2012; Ahy et al., 2020), we excluded individuals either with a history of previously received mindfulness-based intervention or meditation, or with histories of psychiatric disorders.

Baseline characteristics were collected using standardized tablet-based questionnaires administered through face-to-face interviews. Participants were informed that the survey was focused on assessing eating habits. They independently completed the questionnaires, while the researcher was available to address any arising questions and to ensure the thorough completion of the questionnaire. General information regarding infertility duration, type, and obstetric history were extracted from electronic medical records. All methodologies and protocols for data collection were reviewed and approved by the Institutional Review Board of Suzhou Municipal Hospital (No. K-2024-044-K01). Prior to participation, all included individuals were briefed on the study’s purpose and procedures, and provided informed consent at their initial visit to the reproductive center.

### Measure

The Chinese version of Five Facet Mindfulness Questionnaire-Short Form assesses five trait facets of mindfulness which include: observation, description, acting with awareness, non-judgement, non-reactivity. The FFMQ-SF contains 24 items divided into five subscales of either 4 or 5 items. All items are measured on a 5-point Likert scale ranging from 1 (“never or very rarely true”) to 5 (“very often or always true”). Higher scores are indicative of increased endorsement of the mindfulness facets. Previous research demonstrates good psychometric properties (i.e., good internal consistency and construct validity) with the FFMQ-SF (Bohlmeijer et al., 2014).

The symptoms of anxiety were assessed with the GAD-7, a 7-item self-report questionnaire. Scores on the GAD-7 range from 0 to 21, each item is assigned a score of 0 (never) to 3 (every day); cut-offs are 5, 10, and 15 for mild, moderate, and severe anxiety, respectively. The Cronbach’s *α* for the Chinese version is 0.81 (Shan and Li, 2015).

The symptoms of depression were assessed using the PHQ-9, which is based on the nine diagnostic criteria for depression in the Diagnostic and Statistical Manual of Mental Disorders, Fourth Edition, text revision. The PHQ-9 scores range from 0 to 27, cut-offs are 5, 10, 15, and 20 for mild, moderate, moderately severe, and severe depression, respectively. The Chinese version of the PHQ-9 has been validated (Cronbach’s *α* = 0.89) (Kroenke et al., 2001).

Emotional eating was assessed using the Chinese version of the Dutch Eating Behavior Questionnaire (DEBQ-C). The DEBQ-C comprises three domains: emotional eating, external eating, and restrained eating. For this survey, only the emotional eating domain, consisting of 13 items, was utilized. Participants rated the frequency of experiencing these emotions about eating using a Likert scale, with response options ranging from “never” to “always,” scored from 1 to 5, respectively. Scores for emotional eating could range from 13 to 65, with higher scores indicating a greater tendency toward emotional eating. Cronbach’s for the Chinese version is 0.95 (Wu et al., 2017).

### Data analysis

Data were screened to ensure the values of the variables were within the expected ranges and to detect the presence of the possible outliers and the missing data. Descriptive statistics were computed to determine the means, standard deviations, ranges, skewness, and kurtosis for the primary study variables: mindfulness facets (observation, acting with awareness, describing, non-judgment, non-reactivity), depression, anxiety, infertility-related stress, and emotional eating. The Shapiro–Wilk test was used to determine the normality of the data distributions. Pearson correlation coefficients were computed to assess the bivariate relationships among the primary study variables, including mindfulness facets, depression, anxiety, and emotional eating. This preliminary analysis was critical for identifying potential multicollinearity issues and for guiding the selection of variables for inclusion in more complex models. After that, a mediation analysis was conducted to examine whether mindfulness facets mediate the association between depression/anxiety and emotional eating. The indirect effect of depression/anxiety on emotional eating through the different facets of mindfulness was tested. Bootstrapping procedures (with 5,000 resamples) were used to obtain bias-corrected confidence intervals for the indirect effect.

To further investigate the complex relationships among mindfulness, depression, and emotional eating, Structural Equation Modeling (SEM) was conducted using AMOS software. The structural model was specified with observation, acting with awareness, non-reactivity, and non-judgment facets of mindfulness as exogenous variables, depression as a mediating variable, and emotional eating as the endogenous outcome. Model fit indices, such as Comparative Fit Index (CFI), Tucker-Lewis Index (TLI), and Root Mean Square Error of Approximation (RMSEA), were evaluated to assess the adequacy of the model. The SEM analysis provided a comprehensive understanding of the direct and indirect pathways through which mindfulness facets influence emotional eating via depressive symptoms.

## Results

### Descriptive statistics

In the initial sample, 352 participants were recruited for the study. However, due to concerns about data quality, some participants were excluded from the analysis. Specifically, 2 participants were excluded because they reported extreme body mass index (BMI) values, defined as BMI < 17 based on the World Health Organization standards, which indicate extreme thinness and suggest the presence of potential extraneous health conditions that could impact data quality. Additionally, 16 participants were not included in the final analysis due to missing data on more than one item. After these exclusions, a total of 334 participants were included in the data analysis. Please see Table 1 for the baseline characteristics of the participants.

**Table 1:** Baseline characteristics of the participants (*N* = 334).

Characteristic	*N* = 334 (mean ± SD)/ *n* (%)
Age, year (mean ± SD)	30.66 ± 3.68
Education, year	
< 12	112 (33.5)
> 12	222 (66.5)
Household income, CNY (per year)	
< 30,000	26 (7.7)
30,000 -80,000	70 (30.0)
80,000 -150,000	234 (70.1)
> 150,000	4 (1.2)
BMI, kg/m^2^ (Mean ± SD)	24.85 ± 4.85
< 18.5	16 (4.8)
18.5–23.9	100 (29.9)
24–27.9	168 (50.3)
≥ 28	60 (18.0)
Subfertility	
Primary	212 (63.5)
Secondary	122 (36.5)
Parity	
Nulliparous	295 (88.3)
Multiparous	39 (11.7)
Duration of subfertility, years	
< 2	75 (22.5)
2–5	186 (55.7)
> 5	73 (21.9)

BMI, body mass index; CNY, China yuan.

Regression assumptions, including additivity, homoscedasticity, linearity, and normality were examined and scatterplots generated. Collinearity diagnostics revealed that each variable had a tolerance greater than 0.20, showing that each variable has a unique contribution on emotional eating variance, and variance inflation factors that did not exceed 5, indicating that multicollinearity was not a concern. Descriptive statistics including means, standard deviations, minimum and maximum values, range, and bivariate correlations are presented in Table 2.

**Table 2:** Descriptive statistics and bivariate correlation across the primary study variables (*N* = 334).

Variables	Mean	SD	Range	1	2	3	4	5	6	7	8	9
1. PHQ-9	7.44	4.55	27	-								
2. GAD-7	5.71	4.28	21	0.770^**^	-							
3. OB	10.01	3.51	16	0.130^*^	0.167^**^	-						
4. DS	11.76	3.69	16	0.036	0.073	0.430^**^	-					
5. AA	11.04	3.20	16	−0.406^**^	−0.343^**^	0.084	−0.069	-				
6. NJ	10.31	2.82	16	−0.279^**^	−0.287^**^	0.177^**^	0.054	0.414^**^	-			
7. NR	12.17	2.66	16	−0.031	−0.03	0.349^**^	0.426^**^	−0.046	0.009	-		
8. FFMQ	55.29	9.42	58	−0.275^**^	−0.285^**^	0.720^**^	0.664^**^	0.455^**^	0.529^**^	0.566^**^	-	
9. EE	27.56	9.70	52	0.177^**^	0.099	0.236^**^	0.032	−0.208^**^	0.107	0.093	−0.229^**^	-

PHQ-9, Patient Health Questionnaire; GAD-7, Generalized Anxiety Disorder Scale; OB, Observation facet; DS, Describe facet; AA, Acting with awareness facet; NJ, Non-judgement facet; NR, Non-reactivity facet; FFMQ, Five Facets of Mindfulness Questionnaire; EE, Emotional Eating subscale.

* *p* < 0.05; ** *p* < 0.00.

### Correlations among mindfulness facets, depression/anxiety and emotional eating

As shown in Table 2, significant correlations were found among the study variables. Depression (measured by PHQ-9) was positively correlated with emotional eating (*r* = 0.177, *p* < 0.001). Within the mindfulness facets, the observation subscale was positively correlated with both depression (*r* = 0.130, *p* < 0.05) and emotional eating (*r* = 0.236, *p* < 0.001). In contrast, the acting with awareness subscale showed significant negative correlations with both depression (*r* = −0.406, *p* < 0.001) and emotional eating (*r* = −0.208, *p* < 0.001).

### Mediation analysis among mindfulness facets, depression and emotional eating

#### Direct effect of depression/anxiety on emotional eating

Mediation analysis was conducted to examine whether depressive/anxious symptoms mediate the relationship between different mindfulness facets and emotional eating (Table 3). The analysis revealed a significant relationship between depression and emotional eating. Specifically, higher levels of depressive symptoms were associated with increased emotional eating (Coeff = 0.3783, SE = 0.1143, *p* = 0.0010). However, anxiety did not show a significant association with emotional eating.

**Table 3:** Coefficient for depression/anxiety as the mediators of the association between mindfulness and emotional eating (*N* = 334).

	Coeff	SE	*t*	*p*	LLCI	ULCI
Moderator: depression						
Constant	13.461	4.649	2.895	0.004	4.308	22.615
Depression	0.374	0.143	2.613	0.009	0.092	0.656
OB	0.453	0.183	3.029	0.003	0.159	0.748
DS	−0.118	0.188	−0.628	0.530	−0.489	0.252
AA	−0.5464	0.0810	−6.7427	0.0000	−0.7059	−0.3870
NJ	−0.272	0.226	−1.204	0.230	−0.717	0.173
NR	0.189	0.248	0.764	0.446	−0.298	0.677
Moderator: anxiety						
Constant	13.236	4.693	2.820	0.005	3.996	22.475
Anxiety	0.184	0.145	1.267	0.206	−0.102	0.470
OB	0.722	0.185	3.909	<0.001	0.358	1.086
DS	−0.110	0.190	−0.580	0.563	−0.485	0.264
AA	0.614	0.197	3.111	0.002	0.225	1.002
NJ	−0.225	0.229	−0.980	0.328	−0.676	0.227
NR	0.185	0.251	0.737	0.462	−0.309	0.679

Model 1: Depression as mediator between mindfulness facets and emotional eating, *R*^2^ = 0.189, model 2: Anxiety as mediator between mindfulness facets and emotional eating *R*^2^ = 0.124, Covariates included in BMI, age.

OB, Observation facet; DS, Describe facet; AA, Acting with awareness facet; NJ, Non-judgement facet; NR, Non-reactivity facet; FFMQ, Five Facets of Mindfulness Questionnaire; EE, Emotional Eating subscale.

#### Depression as the meditator between mindfulness acting with awareness and emotional eating

Mediation analysis was conducted to examine whether depressive symptoms mediate the relationship between the mindfulness facet of acting with awareness and emotional eating. The direct effect of acting with awareness on emotional eating was found to be significant (Coeff = −0.557, SE = 0.178, *p* = 0.002). Additionally, a significant indirect effect through depressive symptoms was observed (Coeff = −0.207, BootSE = 0.078, LLCI = −0.372, ULCI = −0.068). This suggests that acting with awareness reduces emotional eating both directly and indirectly by lowering depressive symptoms, indicating that depression partially mediates this relationship.

#### Direct effect of mindfulness observation on emotional eating

Results indicated that mindfulness observation had a significant positive direct effect on emotional eating (Coeff = 0.453, SE = 0.183, *p* = 0.003). Mediation analysis showed that depressive symptoms did not significantly mediate the relationship between mindfulness observation and emotional eating. The indirect effect through depression was small and not significant (Coeff = 0.009, LLCI = −0.044, ULCI = 0.075), indicating that the positive association between mindfulness observation and emotional eating exists independently of depressive symptoms.

#### Path analysis of mindfulness facets related to depression and emotional eating

**Figure 1:**
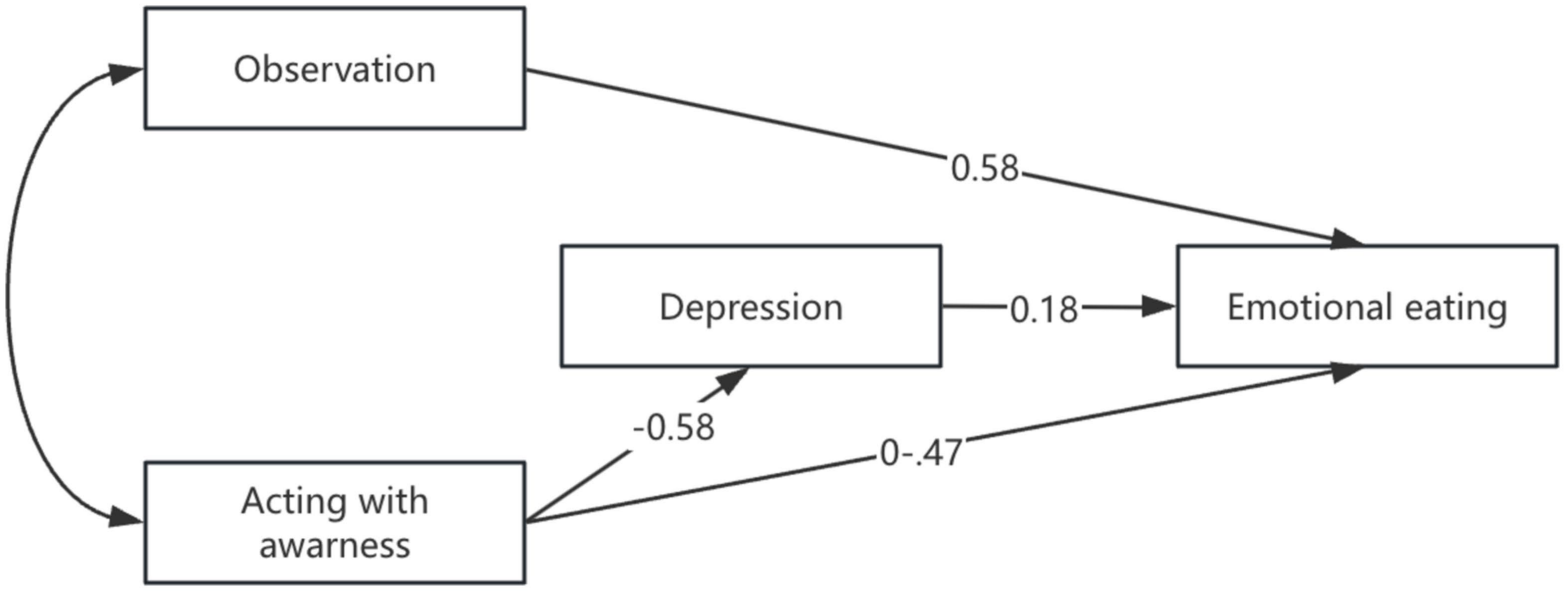
Final model showing the effect of mindfulness facets (observation, acting with awareness) on emotional eating via depression.

Path analysis showed that some of the hypothesized paths were not significant (e.g., among non-reacting/non-judgement to emotional eating and depression) consequently, these paths related to these two variables were removed from the model. The revised model was tested and the results are presented in Figure 1. The revised model showed significant path coefficients ranging from 0.18 to 0.59 and an acceptable fit to the data (CFI = 0.97, NFI = 0.96, RMSEA = 0.068). The bootstrapping procedure confirmed significant direct effects of the observation (confidence interval: 0.113 to 0.312) and acting with awareness (CI: −0.24 to −0.05) on emotional eating. Additionally, acting with awareness was associated with emotional distress indirectly through depression (CI: −0.15 to −0.03).

## Discussion

This study is conducted to explore the association among different facets of mindfulness, depressive symptoms, and emotional eating among individuals with PCOS and infertility. The findings provide new insights into how specific mindfulness traits, specifically observation and acting with awareness regulate emotional eating behaviors by influencing depressive symptoms.

The current study found that the mindfulness facet of acting with awareness was negatively correlated with emotional eating and significantly associated with lower levels of depressive symptoms, which indirectly helped reduce emotional eating. Acting with awareness refers to the ability to stay focused and fully engaged in present activities, rather than operating on autopilot or in a distracted state (Sørensen et al., 2018). This finding aligns with previous researches showing that acting with awareness helps individuals manage emotions and behaviors, thereby reducing emotional eating (Barnhart et al., 2021). Furthermore, an intervention targeting emotional eating through acting with awareness was tested among individuals who self-identified as having difficulties with emotional eating and the positive outcomes suggest that incorporating acting with awareness training could effectively address psychological factors that contribute to overeating (Lattimore, 2020).

Although the mechanisms through which mindfulness regulates emotional eating remain incompletely understood, existing literature has emphasized its role in moderating or interacting with depressive symptoms (Höppener et al., 2019; Barnhart et al., 2021). Consistent with this finding, the current study identified depression as a significant mediating factor in the relationship between mindfulness and emotional eating. However, the effect of depression on the relationship between acting with awareness and emotional eating was found to be only medium-to-low, in contrast to the stronger medium-to-high effect reported in previous studies. This discrepancy may be due to the unique characteristics of individuals with PCOS and infertility, whose emotional eating behaviors are not solely driven by difficulties in emotional regulation. Other factors, such as hormonal fluctuations and anxiety related to body image (Krug et al., 2019), may also play a role in emotional eating, potentially weakening the direct impact of acting with awareness.

Aside from acting with awareness, several studies have found that higher levels of non-reactivity mindfulness can strengthen the relationship between emotional eating and the severity of depression (Barnhart et al., 2021; Hsu and Forestell, 2021; Verrier and Day, 2022). However, in this study, no significant association was observed between non-reactivity and these factors. This discrepancy may be attributed to differences in the sample populations, as our study specifically focused on individuals with PCOS and infertility, who face distinct psychological and physiological challenges compared to other groups (Stener-Victorin et al., 2019). Notably, the average depression score among participants in this study was 7.44, indicating that a significant proportion of individuals experienced depressive symptoms.

In contrast, our study found that the observation facet of mindfulness was positively correlated with emotional eating. Previous research suggests that individuals with PCOS tend to exhibit heightened sensitivity to their bodies. Women with PCOS often experience greater body dissatisfaction compared to those without the condition (Davitadze et al., 2023), and this excessive focus on bodily changes, such as weight gain and other physical symptoms, has been linked to the development of disordered eating behaviors (Burnatowska et al., 2023). Our findings indicate that heightened awareness, as measured by the observation facet of mindfulness, may exacerbate maladaptive behaviors like emotional eating, particularly in individuals who have not received formal emotion regulation or mindfulness training. Without the skills to manage their heightened awareness, these individuals may struggle to differentiate between physical and emotional hunger cues. This aligns with the Monitoring and Acceptance Theory (MAT), which suggests that without proper training, increased awareness can lead to an excessive focus on bodily sensations, making it difficult to distinguish between emotional and physical hunger, thereby potentially triggering unhealthy eating behaviors (Lindsay and Creswell, 2017). Additionally, a longitudinal study on the relationship between mindfulness facets and disinhibited eating also found that higher levels of observation predicted increased external and emotional eating over time (Sala and Levinson, 2017). Therefore, interventions focusing on managing body awareness and emotions should be explored further in this population.

This study has several limitations that should be acknowledged. First, the cross-sectional design limits the ability to determine cause and effect between mindfulness, depression, and emotional eating, therefore future longitudinal studies are needed. Second, the reliance on self-reported data may introduce biases like social desirability and recall errors, despite using validated questionnaires. Third, the study sample, drawn from individuals with PCOS and infertility at a reproductive center in Suzhou, may limit generalizability to broader populations. Additionally, excluding participants with extreme BMI or missing data could introduce selection bias. Lastly, other factors like diet, exercise, or additional psychological variables were not included and should be considered in future research to provide a more complete understanding.

## Conclusion

This study enhances the understanding of how mindfulness relates to emotional eating and depressive symptoms in individuals with polycystic ovary syndrome (PCOS) and infertility. The findings indicate that acting with awareness is negatively correlated with emotional eating and associated with lower depressive symptoms, while observation is positively related to emotional eating. These findings underscore the importance of incorporating mindfulness-based interventions that focus on both acting with awareness and managing bodily sensations. In addition, future research should explore these relationships longitudinally and include a broader range of factors to deepen the understanding of how mindfulness can effectively address emotional eating in diverse populations.

## Data Availability

The original contributions presented in the study are included in the article/supplementary material, further inquiries can be directed to the corresponding author.
